# Virus Enrichment for Single Virus Infection by Using 3D Insulator Based Dielectrophoresis

**DOI:** 10.1371/journal.pone.0094083

**Published:** 2014-06-11

**Authors:** Taisuke Masuda, Hisataka Maruyama, Ayae Honda, Fumihito Arai

**Affiliations:** 1 Department of Micro-Nano Systems Engineering, Graduate School of Engineering, Nagoya University, Chikusa-ku, Nagoya, Japan; 2 Department of Frontier Bioscience, Hosei University, Koganei, Tokyo, Japan; Centers for Disease Control and Prevention, United States of America

## Abstract

We developed an active virus filter (AVF) that enables virus enrichment for single virus infection, by using insulator-based dielectrophoresis (iDEP). A 3D-constricted flow channel design enabled the production of an iDEP force in the microfluidic chip. iDEP using a chip with multiple active virus filters (AVFs) was more accurate and faster than using a chip with a single AVF, and improved the efficiency of virus trapping. We utilized maskless photolithography to achieve the precise 3D gray-scale exposure required for fabrication of constricted flow channel. Influenza virus (A PR/8) was enriched by a negative DEP force when sinusoidal wave was applied to the electrodes within an amplitude range of 20 Vp-p and a frequency of 10 MHz. AVF-mediated virus enrichment can be repeated simply by turning the current ON or OFF. Furthermore, the negative AVF can inhibit virus adhesion onto the glass substrate. We then trapped and transported one of the enriched viruses by using optical tweezers. This microfluidic chip facilitated the effective transport of a single virus from AVFs towards the cell-containing chamber without crossing an electrode. We successfully transported the virus to the cell chamber (v = 10 µm/s) and brought it infected with a selected single H292 cell.

## Introduction

Conventional analysis of viral functions is performed by using virus-infected cultured cells and this method has been considered as the most precise technique of analysis. This method provides information that is an average result of data generated from all of the cells in the population. However, the physiological state and cell cycle stage of each infected cell is different [1]. In order to quantitatively analyze viral effects, analysis of specific cell that is infected by a single virus is required. To satisfy this requirement, we previously constructed a system for the manipulation of a single virus using optical tweezers [2], [3]. This single-virus infection system enabled the effective transport of a single virus from the periphery towards the cell-containing chamber. Recently this system was used to characterize the difference in influenza virus susceptibility between G1- and S/G2/M-phase cells [4]. However, bio-nanoparticles such as the influenza virus (shape: sphere, diameter: approximately 100 nm) tend to be present in samples at a low concentration, and a low virus number provides a limitation to the method. We therefore fabricated an active virus filter (AVF) that could enrich for viruses and modulate virus distribution, by using a dielectrophoretic force.

A dielectrophoretic force is a force that is exerted on a polarizable particle under conditions of a non-uniform electric field [5], [6], [7]. Dielectrophoretic manipulation and accumulation of micro- and nanoparticles as well as its theoretical background were first advocated by Pohl [8]. Biological particles such as cells, bacteria, macromolecules, DNA and viruses have been extensively studied using this method [9]. There is now considerable effort being directed toward applying dielectrophoresis (DEP) for biomedical and biotechnological applications [10], [11], [12]. DEP has been used to trap and analyze individual cells, immobilize cells in an array format, separate different cell types (e.g. viable from dead cells), detect bacteria and manipulate viruses. Conventional electrode-based systems generate electric field gradients by applying an AC signal across two or more metallic electrodes. These systems typically use a coplanar electrode [13], [14] or an interdigitated castellated microelectrode [15], [16], [17], and trap particles at or near the electrode surfaces. Electrode-based DEP systems have been used in the analysis of various particles for the purpose of concentration of the particles in the samples, and they exhibit high selectivity [18], [19].

**Figure 1:**
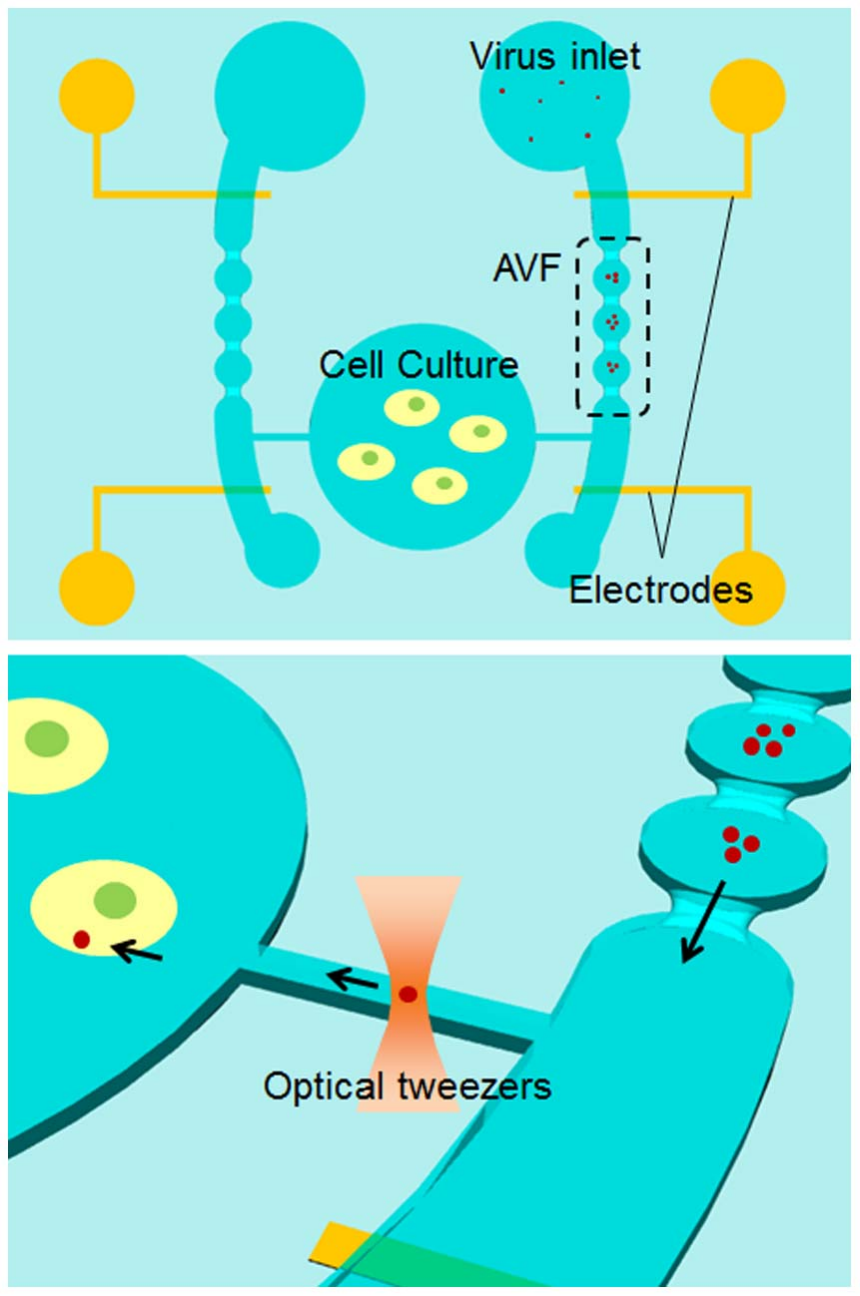
Scheme by which single virus infection of a specific single cell is achieved using an AVF and optical tweezers in a microfluidic chip. A virus (red dot) is selected from an enriched virus population and is then transported to the cell chamber for infection of a specific single cell. There was an advantage of iDEP that optical tweezers can transport without crossing an electrode as compared to standard (microelectrode based) DEP.

In insulator-based dielectrophoresis (iDEP), remote electrodes apply an electric field within a fluidic volume while insulating structures are used to distort the electric field thereby producing spatial non-uniformities [20], [21], [22]. They can be controlled in magnitude by the externally applied signals, the shape of the distribution being defined by the geometry of the insulator [23], [24], [25]. The force generated by iDEP becomes stronger than the force by the conventional DEP at the same input voltage by design of the microchannel. DEP force is dependent to the electric field gradient as shown in equation 1. In conventional DEP, increase of voltage is required to improve DEP force at constant gap between the electrodes. However, input of high voltage induces Joule heating. The generated heat may cause negative effect to the virus and cell in the microfluidic chip. On the other hand, iDEP can increase the DEP force using geometrical design of the electric field gradient. In this research, we fabricated the constricted part in the microchannel to increase the gradient of the electric field in virus enrichment part. Devices for performing iDEP can be made solely from insulating materials (e.g. plastics) that can be cheaply manufactured, which facilitates high-throughput and large-volume applications. An iDEP has been developed for use in the “front-ends” of a filter/concentrator, where it functions as a sensor for bacterial identification, as well as in high-throughput devices that collect bacteria from large volumes of drinking water [20]. On the other hand, Masuda et al. introduced the idea of using insulating constrictions for DEP in microfluidic chip [26]. They microfabricated a constriction with an opening at the center, applied a voltage and trapped and fused a pair of cells. Moreover, arrangement of the electrode is flexible in iDEP. In this paper, single virus is manipulated by optical tweezers. IR laser (wavelength: 1064 nm, TEM00) is used for optical tweezers. The laser power input to the sample is about 100 mW. The diameter of the focused laser is around 2 µm in case of use of objective with high N.A. (1.3). Therefore, the power density of the laser is about 32 kW/mm^2^. The electrode is made of Cr/Au. When the focused laser is irradiated to the electrode, the metal electrode is broke, and the bubble is generated. Therefore, the electrodes must be arranged around working space of enrichment and transport parts. A large gap between electrodes enough to manipulation of the virus without crossing the electrode is possible by using iDEP as shown in Figure 1.

In this study, we present a virus infection microfluidic chip in which three-dimensional iDEP was used generate an AVF that could enrich for and allow manipulation of viruses in an aqueous solution. This system facilitated the effective transport of a single virus from the periphery towards the cell-containing chamber without crossing an electrode. The ultimate goal of this research was to carry out on-chip infection of a specific cell with a single virus using optical tweezers.

## Materials and Methods

### Design of the Active Virus Filter (AVF)

Figure 1 shows the design concept of the microfluidic chip for infection of a specific single cell with a single virus. The microfluidic chip consists of an AVF, which is subjected to iDEP, an analysis chamber for infection of cultured cells, and a microchannel for buffer flow. The iDEP relies on insulating features and a flow channel with a constricted design in the microfluidic chip to create a non-uniform electric field [27]. The virus solution was first loaded into the inlet channel and the buffer was simultaneously injected as a sheath flow. The viruses were concentrated on the AVF by generating an iDEP force, which was generated by adjusting the conductivity of the solution and the frequency of the voltage for negative dielectrophoresis. A single virus was selected from the concentrated group of viruses and this virus was trapped and transported to the cell chamber using optical tweezers. There was an advantage of iDEP that optical tweezers can transport without crossing an electrode as compared to standard (microelectrode based) DEP. The transported virus was then brought into attachment with the target cell to infect it.

### Analyses of the Electric Field of the AVF

DEP is the motion of a particle in the medium in which it is suspended due to the presence of a non-uniform electric field. The dielectrophoretic force acting on a spherical particle can be described by the following equation [28]:
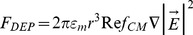
(1)

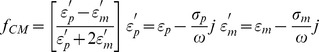
(2)where *E* is the electric field strength, *r* is the particle radius, *ε'_p_* and *ε'_m_* are the respective complex permittivities, *ε_p_* and *ε_m_* are respective permittivities, *σ_p_* and *σ_m_* are the conductivities of the particle and the suspending medium, respectively, and *ω* is the angular frequency of the voltage. Ref_CM_ is the Clausius–Mossotti factor related to the effective polarizability of the particle. When high frequencies are applied and the particles are less polarizable than the suspension medium, the particles exhibit “negative” DEP by moving away from regions of high electric field gradient. iDEP systems use both AC and DC electric fields, therefore, the DEP force exerted on a particle for electric field gradient depends on the dielectric properties of the particle and the suspension medium. The DEP force exerted on a particle also depends on the particle shape in addition to size.

**Figure 2:**
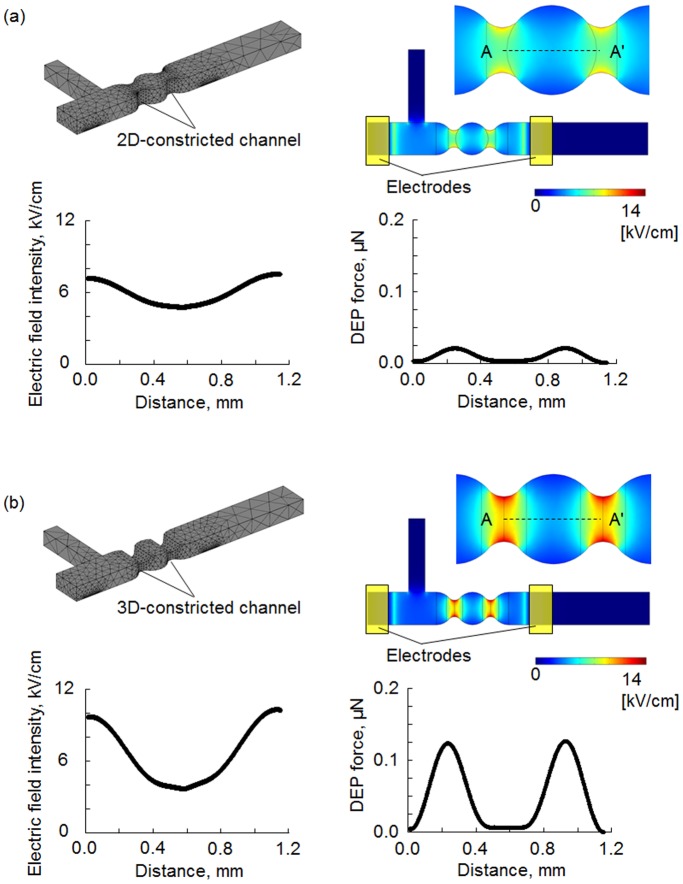
Scheme of the constricted flow channels and FEM analysis of electrical field intensity. The constricted flow channel in the analytical model was provided by either two dimensional (2D) microfabrication (a) or 3D microfabrication (b). A non-uniform electric field with a maximum of 0.11 µN was applied and the electric field intensity (left) and the dielectrophoretic force along the center of the microchannel (i.e., at the indicated distance along the line AA’, right) were measured (Input voltage: 20Vp-p). These data indicated that a channel obtained by 3D microfabrication was better than a 2D-constricted channel.

Figure 2 shows the detailed design of two different active virus filters (AVFs) with the electrodes, and the simulation of electric field distribution in these AVFs that was determined using FEM analysis software (COMSOL Multiphysics 3.5a, COMSOL Inc., Burlington, MA, USA). In addition, this model employed the material properties of the virus and the culture medium solution: *ε_p_ = *3 F/m, *σ_p_* = 0.8 mS/m [9]. This analytic model provided for the generation of a constricted flow channel by 2D microfabrication (Fig. 2-a) or by 3D microfabrication (Fig. 2-b). These channels are hereafter referred to as 2D- or 3D-constricted channel, respectively. As shown in the top panels of Fig. 2-a and -b, two electrodes were fabricated on the glass substrate; the distance between each electrode and the constricted part of the flow channel was 500 µm. When AC voltage (20 Vp-p, 10 MHz) was applied, the maximum electric field strengths around the constricted part of the 2D- and 3D-constricted flow channels were 7.6 and 14.0 kV/cm, respectively. The maximum electric field gradients were 2.8 kV/cm and 6.4 kV/cm. Here, this model employed the material properties of the culture medium solution: *ε_m_ = *80 F/m, *σ_m_* = 10 mS/m. The value of f_CM_ factor was −0.36. Therefore, the virus received negative DEP force. Figure 2 shows the electric field intensity and the dielectrophoretic force along the center of the microchannel (i.e., at the indicated distance along the line AA’) for the 2D- and 3D-constricted channels, respectively. This analysis showed that, using a non-uniform electric field with a maximum of 0.11 µN, the 3D-constricted channel proved better than the 2D-constricted channel. For this reason, three-dimensional microfabrication was considered to be a requirement for construction of the constricted flow channel.

A detailed analysis of the factor *E* is often required for the design of the electrodes in a DEP device. From equation (2), the *ω* is the angular frequency of the applied electrical field *E*. The expression for the DEP force as a function of the distance between the two electrodes is defined as [29], [30];
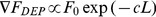
(3)where *F_0_* is the value of *F* when the distance between the two electrodes *L* = 0, and *c* is a constant depending on the configuration of microchannel and electrodes. The DEP force is commonly dependent on the distance between electrodes. Another simulation was therefore performed to investigate the DEP force with respect to the distance between electrodes using a 3D-constricted channel (Fig. S1). Numerical simulations were carried out using COMSOL 3.5a. Simulations were carried out in which the distance between electrodes was set at either 4 mm (*L_1_*) or 7 mm (*L_2_*) and in which single or multiple AVFs were inserted between electrodes set at a distance of 7 mm. The simulation results show that for a planar electrode, the electric field gradient dramatically decays in a cross-section of the microchannel as the distance between electrodes increases. Therefore, the DEP force is very weak for an AVF that is inserted into separated electrodes. One method to increase the DEP force that acts on the particle is to increase the applied voltage. However, an increase in the voltage can result in an increase in the temperature in the microfluidic channel. Intriguingly, the data in Fig. S1 shows that the problem improves a weak DEP force that results from separated electrodes can be enhanced by installing two or more 3D-constricted channels between electrodes. Thus compared with a single AVF, an iDEP chip with multiple AVFs provides a more accurate and faster method of improving the trapping efficiency of viruses. Furthermore, by using multiple AVFs reduction in error creation is expected, since bonding alignment with an electrode and a microchannel is easier than with a single AVF.

Moreover, DEP force is used to prevent the adhesion of the virus to the glass substrate. In the microchannel, forces working to the virus are gravity force, DEP force and Brownian force. The virus does not adhere to the glass in case the DEP force exceeds the Brownian force and gravity force. The Brownian force is given by:
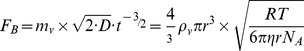
(4)where *F_B_* is the Brownian force [N], *ρ_v_* is the virus density [g/cm^3^], *r* is the virus radius [m], *R* is the molar gas constant (8.31447 J K^−1^ mol^−1^), *T* is the temperature [K], *η* is the viscosity of the solution (1.004×10^−6^ m^2^/s), and *N_A_* is the Avogadro constant [/mol]. We assumed that the virus density was 1 g/cm^3^, and time resolution *t* is 0.33 s. From Eq. 4, *F_B_* was 8.3×10^−24^ N. Gravity force of the virus *F_G_* is 5.1×10^−18^ N. When *F_DEP_* is larger than *F_B_ +F_G_*, the virus inside the microchannel does not adhere to the glass during enrichment of virus by DEP force.

### Fabrication of the AVF

**Figure 3:**
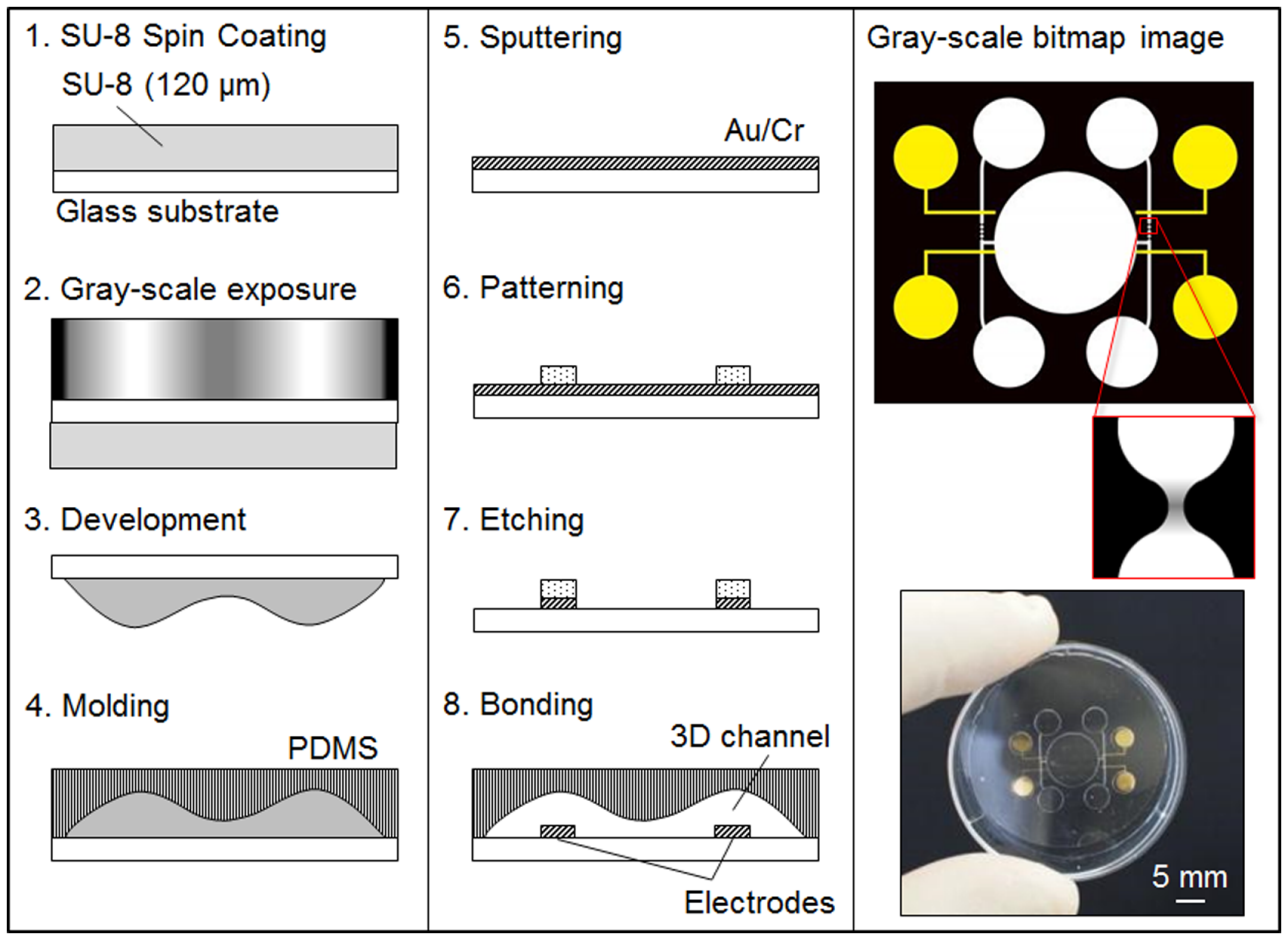
Fabrication process of the 3D microstructure of the constricted flow channel using maskless gray-scale lithography. The devices shown are polymer microfluidic chips made using PDMS that were injection molded using photolithography and replica molding techniques. The chip mold was made using a maskless exposure system that achieved synchronous fabrication of the micropattern in the displayed images that were generated by a PC. The light images shown at right are the micropattern image of the maskless photolithography device (top) and a gray-scale exposure (bottom).

Three-dimensional (3D) patterns whose shape is precisely controlled can be fabricated using gray-scale lithography. Gray-scale lithography utilizes locally modulated exposure doses to develop a 3D structure in a photoresist [31], [32], [33]. Differential exposure doses lead to a photoresist that is exposed at multiple depths across its surface. Gray-scale photolithography is a promising technique for achievement of a precisely controlled 3D structure based on exposure dose, because the profile of the exposure dose can be easily controlled by changing the profile of the contrast of the gray-scales. However, gray-scale lithography requires specialized equipment and preparation of photomasks, resulting in a costly and time-consuming process. An alternative technique is to use a maskless exposure system, which does not require hard masks [34], [35]. This maskless exposure system achieves synchronous fabrication of a micropattern in the displayed images that are generated by a PC through an LCD projector. Figure 3 shows the process by which the microfluidic chip was fabricated to create a 3D constricted flow channel by using maskless gray-scale lithography. First, a g-line negative photoresist (SU-8, 3000cp; Tokyo Ohka Kogyo Co. Ltd., Kawasaki, Japan) is spin coated on a glass substrate (about 150 µm thick) (1) and then a gray-scale bitmap image is utilized to obtain a 3D photoresist pattern (2). The photoresist is removed in the developer solvent (3) and the 3D photoresist pattern is transferred into polydimethysiloxane (PDMS) (Silpot 184, Dow Corning Toray, Co. Ltd., Tokyo, Japan) (4). For the fabrication of electrodes, an Au/Cr layer is sputtered onto a glass substrate (5). A positive photoresist (OFPR, 200cp; Tokyo Ohka Kogyo Co. Ltd., Kawasaki, Japan) is then spin coated and patterned to fabricate the electrodes (6). After development, the Au/Cr layer is etched by individual etchant (7). Finally, the obtained PDMS channel is treated with oxygen plasma to increase its adhesion to with the electrode glass substrate. (8).

A photograph in Fig. 3 shows a virus infection of H292 cells using a microfluidic chip and an AVF. Three-dimensional surface profiles were obtained with a microfigure measuring instrument (SURFCORTER, ET-200S; Kosaka Laboratory Ltd., Tokyo, Japan). The depth and width of the main channel are 150 µm and 150 µm, respectively. Those of constricted channel are 50 µm and 50 µm, respectively. The diameter of the analysis chamber is 8 mm. Width and gap of the electrodes are 150 µm and 4 mm, respectively. An Au/Cr electrode pattern was fabricated in the flow channel that was connected with the AVF. By using gray-scale lithography, the height of the constricted flow channel descended toward the bottom at 80 µm from the main channel. The depth of the constricted flow channel could be controlled within a range of 1–100 µm by using the gray-scale calibration of SU-8 (Depth resolution is about 1 µm).

### Enrichment of the Influenza Virus on the AVF

The influenza viruses (A PR/8) were co-stained with 1,1′-Dioctadecyl-3,3,3′,3′-tetramethylindocarbocyanine Perchlorate (DiI) (Molecular Probes; Ex 549 nm, Em 565 nm) and SYTO 21 live-cell nucleic acid stain (Molecular Probes; Ex 494 nm, Em 517 nm) which stain the virus membrane and virus ribonucleoprotein (RNP), respectively [4]. Human lung mucoepidermoid carcinoma cells (H292 cells) were cultured in Dulbecco’s modified Eagle’s medium (DMEM) (Wako, Osaka, Japan) with 10% FBS. The H292 cells were pre-cultured in the cell chamber of the microfluidic chip before virus enrichment. The microfluidic channel and a cell chamber were then filled with a buffer solution containing 10 mM HEPES (pH 7.6). The conductivity of this solution was adjusted to a value of 10 mS/m by adding 10 mM KCl. The flow rate was 0.10 µl/h (flow speed in the trapped area: 5 µm). A high-frequency (20V_p-p_, 10 MHz) voltage was applied to the electrodes by a function generator (WF1974, NF Co., Yokohama, Japan). We used a confocal laser microscope (CSU-X1, Yokogawa Electric Co., Tokyo, Japan) equipped with a laser manipulation system (maximum power: 1 W; output wavelength: 1064 nm) to perform iDEP virus enrichment and single virus infection of a specific cell. The virus was tracked through the process of iDEP, transport and infection by visualization of viral fluorescence using the same microscope. The cells of influenza virus attached and unattached cells were assayed by immunostaining using anti-PB1. At 4 h after virus attachment on the cell, the cells were washed with PBS and fixed with 4% paraformaldehyde solution, treated with 0.5% Triton X-100, blocked with 1% BSA. After blocking, the cells were incubated with anti PB1 antiserum for 1 h, washed with 1% BSA/PBS solution and then incubated with anti-rabbit IgG with Cy3 for 1 h at 37°C [4].

### Laser Manipulation System for Single Virus Transport

We used inverted microscope (Ti-E, Nikon Co., Tokyo, Japan) having a laser confocal scanning system (A1R, Nikon Co., Tokyo, Japan) for fluorescence observation and a laser manipulation system (laser wavelength: 1064 nm, maximum laser power: 2 W, Sigma co. Ltd.) for virus manipulation. The laser is focused by objective lens (Magnification: 100) with high numerical aperture (NA: 1.3). The virus is trapped at the laser focal point by Lorentz force because the size of the virus (100 nm) is smaller than that of laser wavelength. Trapping force becomes bigger according to the increase of laser power. However, laser power is limited within 1 W to avoid photo-bleaching of Syto 21 during manipulation. In fact, even if laser power output 1 W, input to a sample is decreased to about 100 mW since an objective lens is passed. In this paper, all transport speed of the virus is 10 µm/s.

## Results and Discussion

**Figure 4:**
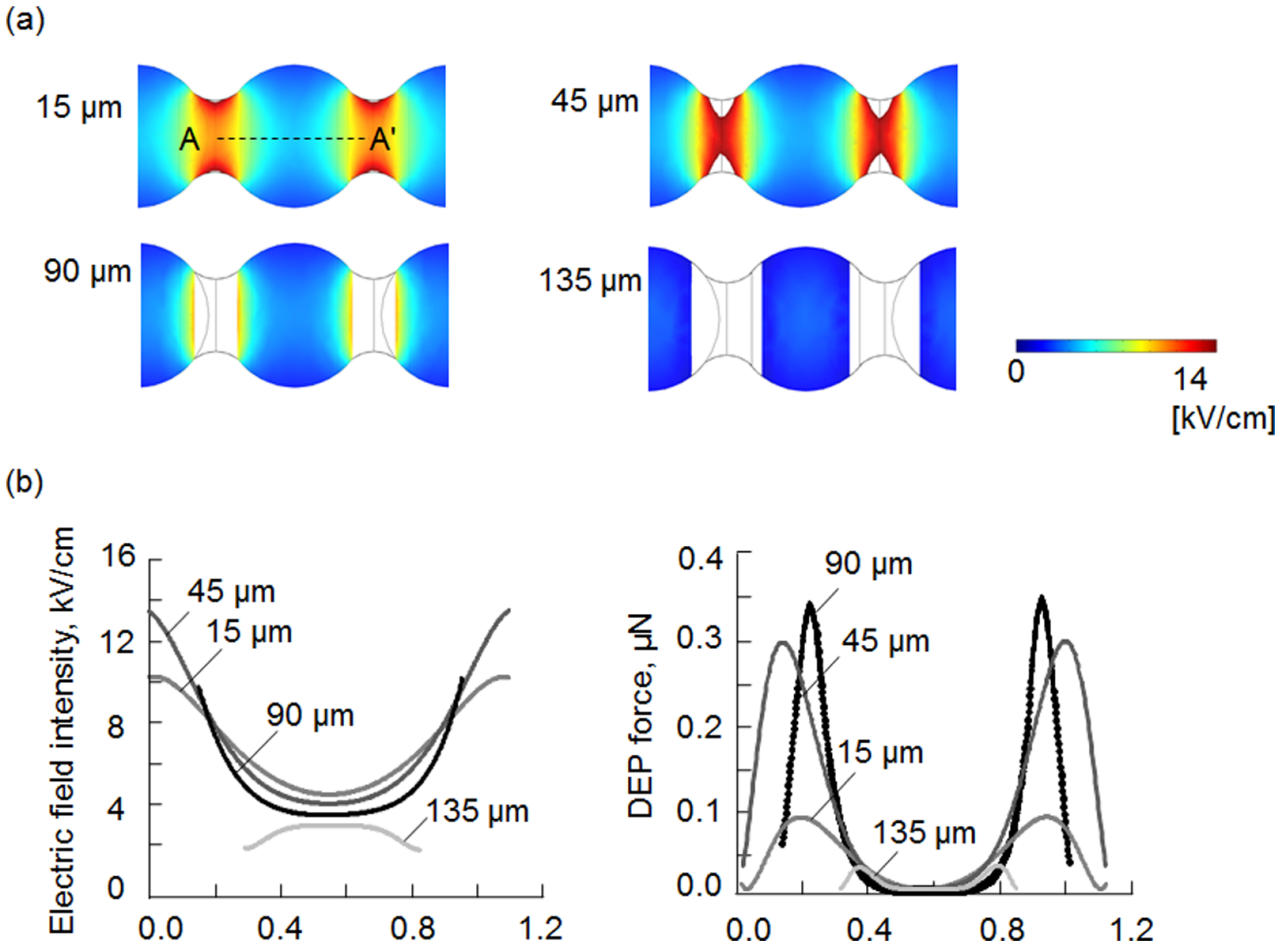
Simulation results of the electric field distribution of an active virus filter (AVF) with height channels. Analysis of the active virus filter by FEM (a), the electric field distribution intensity in microchannels at heights of 15, 45, 90 and 135 µm from the glass bottom. (b) The electric field intensity and the dielectrophoretic (DEP) force along the centre of the microchannel (i.e., at the indicated distance along the line AA’) at each height are shown.

Figure 4-a) shows the simulation results of the electric field distribution of an active virus filter (AVF) with channels whose heights were constricted to 15, 45, 90 or 135 µm. The white region represents PDMS microchannel. When 20 Vp-p was applied, the values of the maximum electric field strength around the constricted part of these 3D multi-constricted flow channels were 14.0, 17.9, 10.1 and 0.5 kV/cm, respectively, with a high gradient. Fig. 4-b) shows the electric field intensities and the dielectrophoretic force along the center of the microchannel (i.e., at the indicated distance along the line A-A’) at each height. The conductivity and permittivity of the influenza virus that was employed in this work was previously reported by Schnelle et al. as 0.8 mS/m and 3 F/m [9], respectively. By employing Eq. (2), it is possible to calculate the dielectrophoretic force at each analytic surface. Analysis of the data shown in Fig. 4-b) indicates that the maximum *F_DEP_* of the four analytic surfaces can be ordered according to the constricted microchannel height as follows: 135 µm <15 µm <45 µm <90 µm. The reason why the gradient generation in each height is considered that by increasing the channel height, the gradient of the electric field should decrease as the electric field streamlines are less disturbed at the trapping area. The highest iDEP power observed for the four analytic surfaces was that of 0.3 µN that was observed at a constricted channel height of 90 µm. In the previous study, we estimated the force for transporting the virus by optical tweezers using Stokes’ law. When the size of influenza viruses is 100 nm, the driving force was calculated to be 9.4 fN [3]. Therefore, we decided to turn off an AVF in a single virus transporting since the negative DEP force exceeds the driving force by optical tweezers.

**Figure 5:**
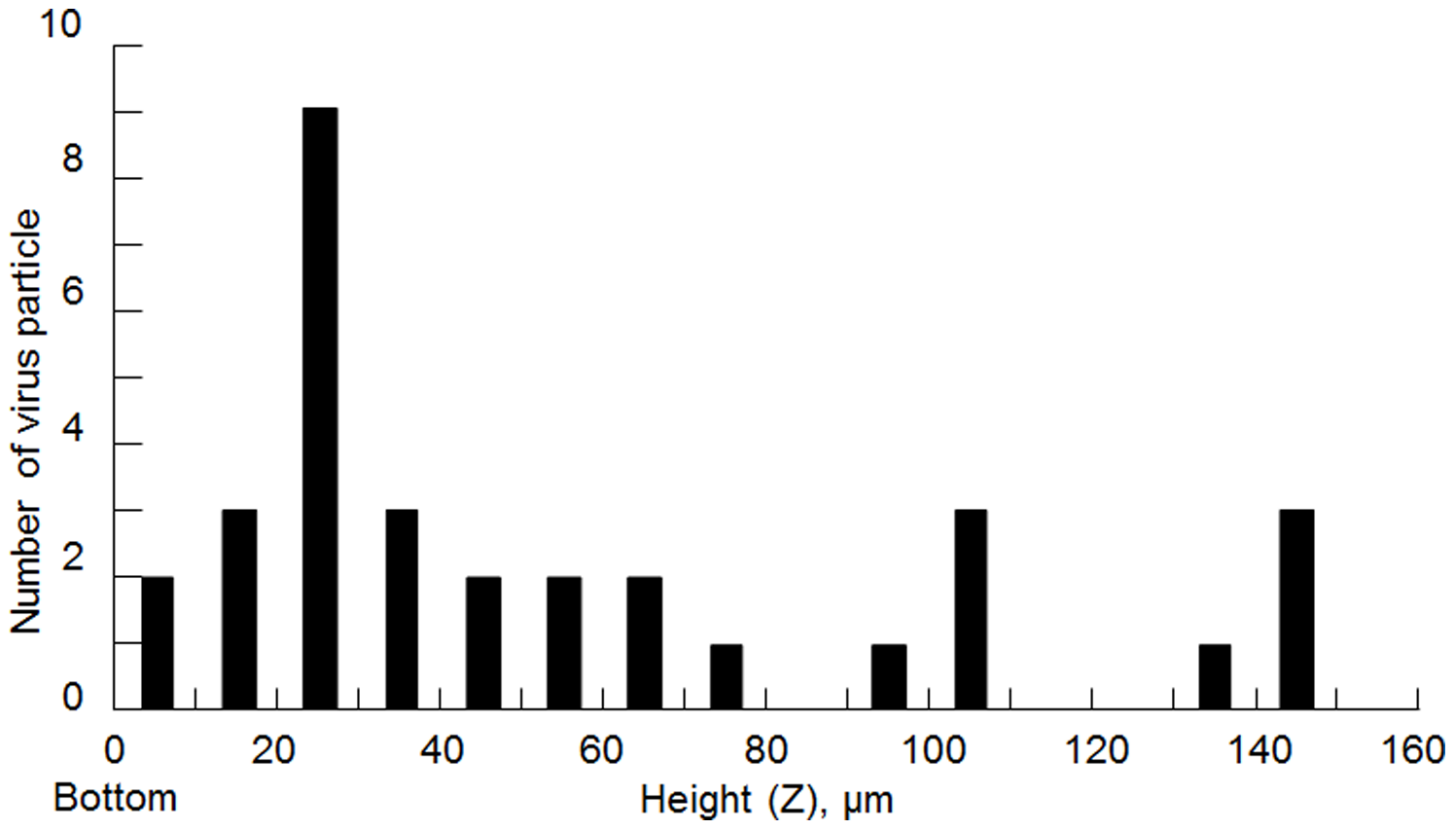
Distribution of the virus at various heights from the bottom of the microfluidic channel. A large number of viruses was trapped at heights of about 10–30 µm from the glass substrate. The AVF inhibits adhesion of the virus to the glass substrate.

In experiment, virus solution (concentration: 10^6^ pfu/mL) was introduced into the AVF. The introduced viruses were enriched by iDEP (voltage: 20 V_p-p_, frequency; 10 MHz, sine wave) in AVF during 10 sec. AVF-mediated virus enrichment can be repeated simply by turning the current ON or OFF as showing Movie S1. After enrichment, we then determined the total number of fluorescent influenza viruses in each section by confocal microscopy Z-scan imaging using 1500 z-sections with z-scan steps of 100 nm. Figure 5 shows the experimental result of the distribution of the viruses at different heights in the microfluidic channel after enrichment. Here, the surface of glass substrate is a bottom (height = 0). The data in this figure indicate that the viruses exhibit negative dielectrophoretic behavior. Furthermore, the viruses were observed to be trapped at regions of the channel that were at a distance from the glass bottom, since they were trapped closer to the region of the high electric field gradient. As a result of observing around this region using a confocal microscope, about 200 times as much virus concentration was shown. The viruses were trapped at heights of up to about 10–30 µm from the glass substrate. During the enrichment, convection flow due to the joule heat did not occur in these conditions. Thus, the use of an AVF appears to inhibit adhesion of the viruses to the glass substrate. These results demonstrated that AVF has dual functions of virus trapping and inhibition of virus adherence to the glass substrate.

**Figure 6:**
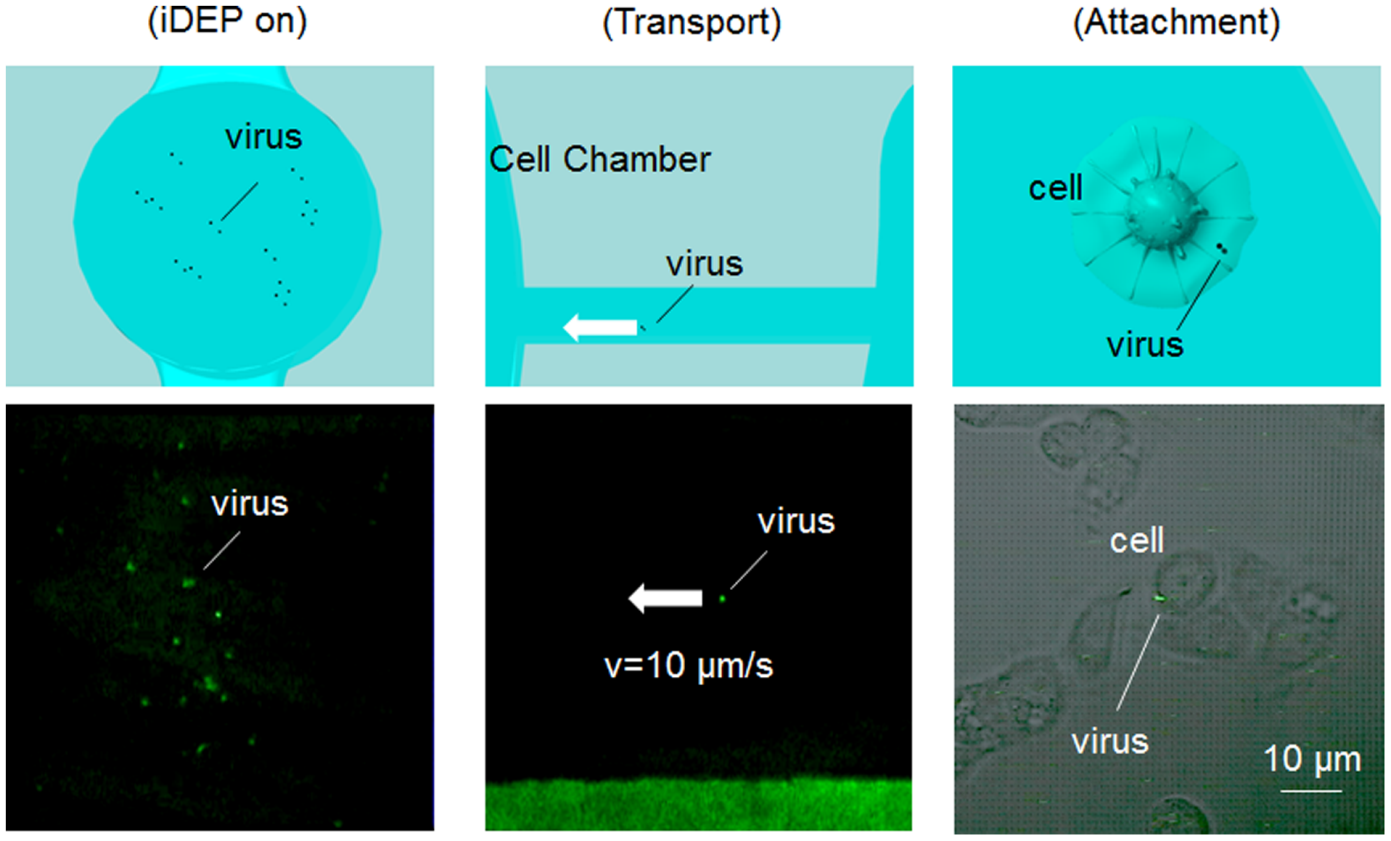
Enrichment, transport and attachment of influenza virus. Enrichment of the influenza virus in the AVF by a negative DEP force, transport of a single virus to the cell chamber by using optical tweezers and attachment with a selected H292 cell by the virus. The process of iDEP, virus transport and viral infection of an H292 cell is outlined in the top panels. The virus was tracked through these processes by visualization of the green fluorescence of a virus that was co-stained with DiI and SYTO 21 (bottom panels) using confocal microscopy.

**Figure 7:**
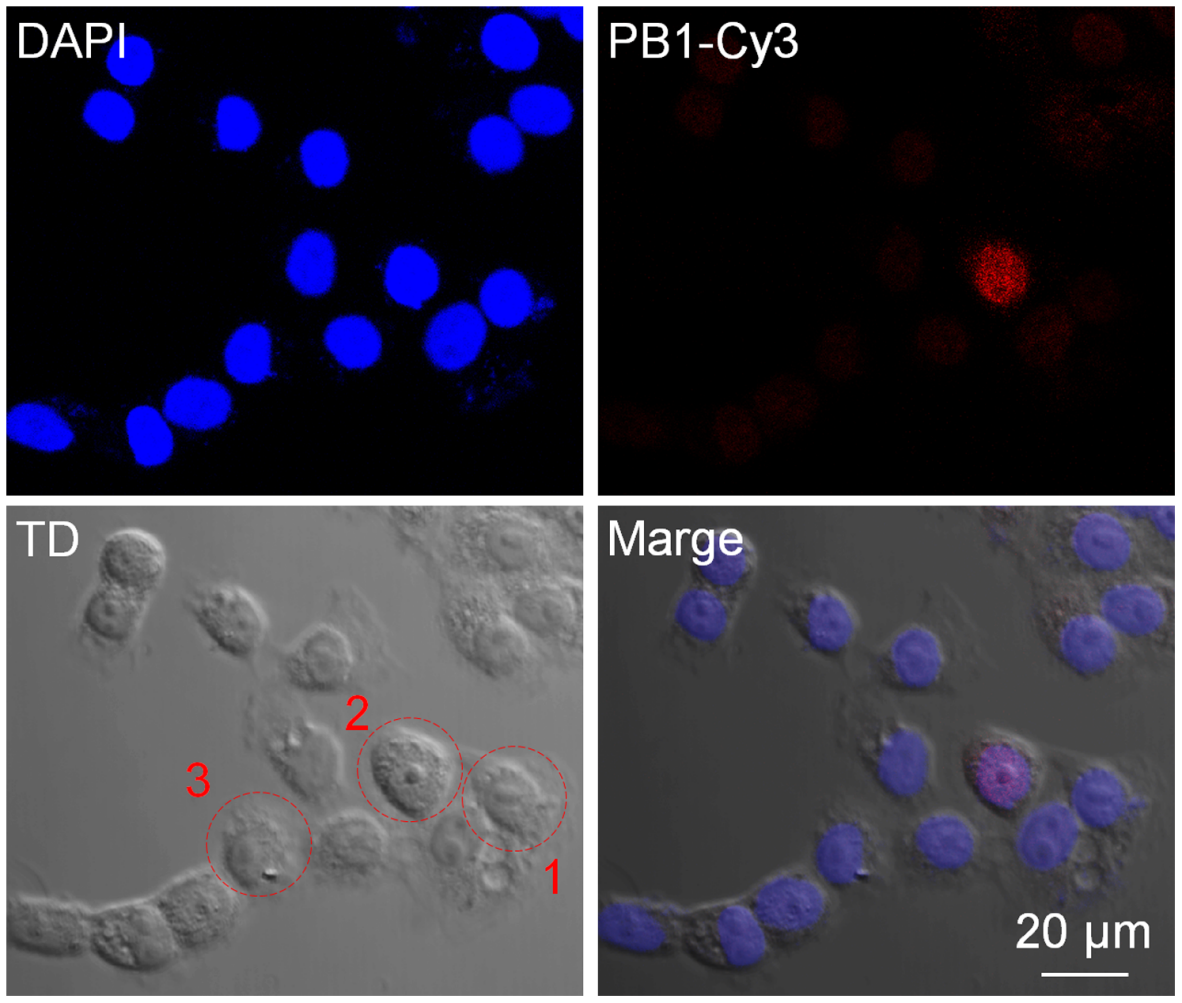
Infection of a virus to the selected H292 cell. Fluorescent images of cells at 4-PB1 antiserum and anti-rabbit IgG conjugated Cy3 (Red). Nucleus, staining with DAPI (Blue).

We succeeded in trapping one of the enriched viruses by using optical tweezers. Finding and trap of single virus is difficult because the virus is quite small. Depth of the focus is less than 1 µm in use of the objective lens (NA: 1.3). Moreover, Brownian motion of the virus is fast and the virus moves away from the focus plane easily. Therefore, enrichment of the virus by AVF is quite useful by DEP to find the virus before laser transport phase. Moreover, we transported this virus to the cell chamber where it made attachment with the selected H292 cell for infection (Fig. 6 and Movie S2). Infection was confirmed by detection of the PB1 subunit of influenza virus by immunostaining of the cell using anti-PB1 serum at 4 h after virus attachment. As shown in Figure 7, we succeeded in infection of a virus to the selected H292 cell. However, the microscopic observations indicated that the infection of influenza virus particles to H292 cells was not uniform, suggesting the cell cycle-dependent variation in virus susceptibility. Our previous study indicated that influenza virus binds preferentially to G1-phase cells with higher level of sialic acid content. It is reasonable to think that difference of cell cycle could have an effect on infection virus to three cells.

We should note that electrode allocation of iDEP contributed to the safe and easy transportation of viruses using optical tweezers. Our previous study indicated that the direct irradiation of laser was caused since the pectinate electrodes and transportation path crossed [3]. On the other hand, since there are no electrodes located across this transportation path in this study, we can avoid direct irradiation of laser by the electrode. Therefore, this microfluidic chip facilitated the effective transport of a single virus from AVFs towards the cell-containing chamber without crossing an electrode. This is the greatest advantage of using iDEP. A further important point is that iDEP traps have a high flexibility for the design of a microchannel. As the FEM results, iDEP can determine the position of maximum electric field gradients by the design of a microchannel. The viral-cell infection process is a very intriguing interaction [36]. It starts with attachment between the virus and the cell membrane and finally results in transport of the virus into the nucleus and gene expression. It is essential to understand these processes for antiviral drug design, as well as for the development of efficient gene therapy vectors. We designed the microfluidic chip presented here to selectively enrich for viruses using iDEP in order to enable single virus infection of a specific single cell. We demonstrated the feasibility of this active virus filter with iDEP for reliable enrichment and distribution of a virus. An additional advantage of this technology is that we utilized maskless photolithography to achieve precise 3D gray-scale exposure at a low cost. AVF can be quickly turned on or off without a decrease in performance. Since this filter can perform virus enrichment and distribution at will, it will therefore contribute to the future of quantitative analysis of viruses and viral functions.

## Conclusion

We developed an active virus filter (AVF) that enabled virus enrichment and distribution for single virus manipulation by using 3D insulator-based dielectrophoresis (iDEP). The design of the 3D constricted flow channel enabled the microfluidic chip to produce an iDEP force. We utilized maskless photolithography to achieve precise 3D gray-scale exposure for construction of the constricted flow channel. The use of the AVF achieved enrichment of the influenza virus via a negative dielectrophoretic force. Negative AVF also functions to inhibit virus adhesion onto the glass substrate due to 3D iDEP effect. In this result, we succeeded in infection of a virus to the selected H292 cell.

## Supporting Information

Figure S1 **Investigation of the relationship between the DEP force and the distance between electrodes by FEM analysis of electric field distribution.** Simulations were carried out in which the distance between electrodes was either 4 mm (*L_1_*) or 7 mm (*L_2_*). The effect of single or multiple AVFs between electrodes set at a distance of 7 mm was also analyzed. The results of FEM analyses are shown.
(TIF)

Movie S1 **Enrichment of influenza virus in AVF.** Movie showing the iDEP enrichment of influenza virus around the constricted flow channel. We applied sinusoidal wave to the electrodes within the range of amplitude of 20 Vp-p and frequency of 10 MHz. AVF achieved enrichment of influenza virus by the negative dielectrophoretic force repeatedly.
(MP4)

Movie S2 **Observation by which an influenza virus is attached to a specific cell by optical tweezers.** Movie showing the attachment of a SYTO 21-labeled influenza virus particle to three H292 cells following manipulation with optical tweezers.
(MP4)
