# Supplementary figures and images for: Virus Enrichment for Single Virus Infection by Using 3D Insulator Based Dielectrophoresis
Source: PLoS One. 2014 Jun 11;9(6):e94083. doi: 10.1371/journal.pone.0094083 (PMC4053322; doi:10.1371/journal.pone.0094083)

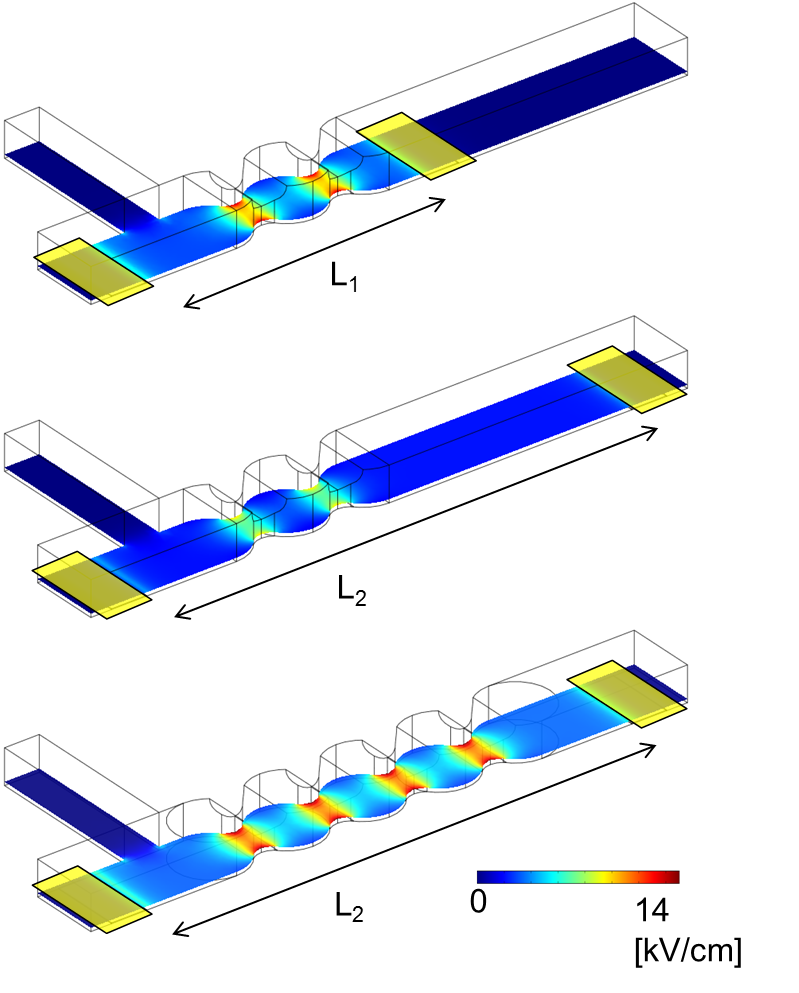

Supplement: Figure S1 — Investigation of the relationship between the DEP force and the distance between electrodes by FEM analysis of electric field distribution. Simulations were carried out in which the distance between electrodes was either 4 mm (L1) or 7 mm (L2). The effect of single or multiple AVFs between electrodes set at a distance of 7 mm was also analyzed. The results of FEM analyses are shown. (TIF) [file pone.0094083.s001.tif]
